# Glycoside fraction from pear seed functionally antagonizes sFLT-1 and restores angiogenic signaling in preeclampsia

**DOI:** 10.1038/s41440-026-02770-x

**Published:** 2026-09-01

**Authors:** Peter U. Amadi, Justice O. Osuoha, Atieme J. Ogbolosingha, Govind S. Gill, Suha J. Jarad, Prince C. Odika, Esienanwan E. Efiong, Sayem A. Ahmed, Emmanuel N. Agomuo, Queendarlyn Agbagwara, Chiamaka W. Amadi, Joy A. Amadi, Celestine N. Ekweogu, Hong-mei Gu, Da-wei Zhang

**Affiliations:** 1 Department of Pediatrics, Faculty of Medicine and Dentistry, University of Alberta, Edmonton, AB Canada; 2 Department of Biochemistry, Imo State University, Owerri, Nigeria; 3 School of Chemistry, Monash University, Clayton, VIC Australia; 4 Department of Science Laboratory Technology, Imo State Polytechnic, Omuma, Nigeria; 5 Department of Life Sciences, Imperial College London, London, UK; 6 Department of Biochemistry, Faculty of Medicine and Dentistry, University of Alberta, Edmonton, AB Canada; 7 Department of Biology, St. Mary’s University, Halifax, NS Canada; 8 Department of Biochemistry, Federal University of Lafia, Lafia, Nigeria; 9 Department of Nutrition, Imo State University, Owerri, Nigeria; 10 Department of Medical Biochemistry, Imo State University, Owerri, Nigeria

**Keywords:** Endothelial dysfunction, Glycosides, implemental hypertension, morning hypertension, Preeclampsia

## Abstract

Preeclampsia is characterized by endothelial dysfunction and angiogenic imbalance driven, in part, by excess soluble fms-like tyrosine kinase-1 (sFLT-1). Building on previous findings demonstrating vascular modulatory effects of cardiac and quercetin glycosides from Dacryodes edulis seeds during L-NAME-induced vascular perturbation, we investigated whether pear seed glycosides (PSG) modulate sFLT-1-mediated endothelial injury in preeclampsia. Endothelial, preclinical, biophysical, pharmacodynamic, and exploratory clinical approaches were integrated to evaluate PSG activity. TGFβ-stimulated HUVECs, an L-NAME-induced rat model of preeclampsia, orthogonal binding assays (SPR/BLI and MST), ligand competition assays, and a multicenter double-blind phase 1-2a exploratory clinical trial were employed. PSG reduced endothelial migration and suppressed αSMA, TNFα, and fibronectin expression in stimulated HUVECs. In preeclamptic rats, PSG attenuated systolic and diastolic blood pressure elevation, reduced inflammatory and cardiovascular injury markers, restored VEGF and placental growth factor (PlGF), and reduced circulating sFLT-1 and the sFLT-1/PlGF ratio. Bioactivity-guided fractionation identified glycosides as the principal active fraction and improved mean birth weight from 2.7 g in untreated preeclamptic dams to 3.5 g, approaching normotensive controls (3.7 g). Orthogonal SPR/BLI and MST assays demonstrated concentration-dependent PSG-sFLT-1 interactions, while competitive ELISA and BLI assays showed inhibition of sFLT-1-mediated sequestration of VEGF and PlGF. PSG additionally restored VEGFR2/KDR, AKT, and ERK signaling in sFLT-1-exposed endothelial cells. In the exploratory clinical trial, 83 participants were randomized following screening of 125 women, with approximately 80% completing follow-up. PSG administration alongside standard care did not reveal major treatment-emergent safety concerns. These findings support further investigation of PSG as a candidate adjunctive strategy targeting angiogenic dysfunction in preeclampsia.

**Figure:**
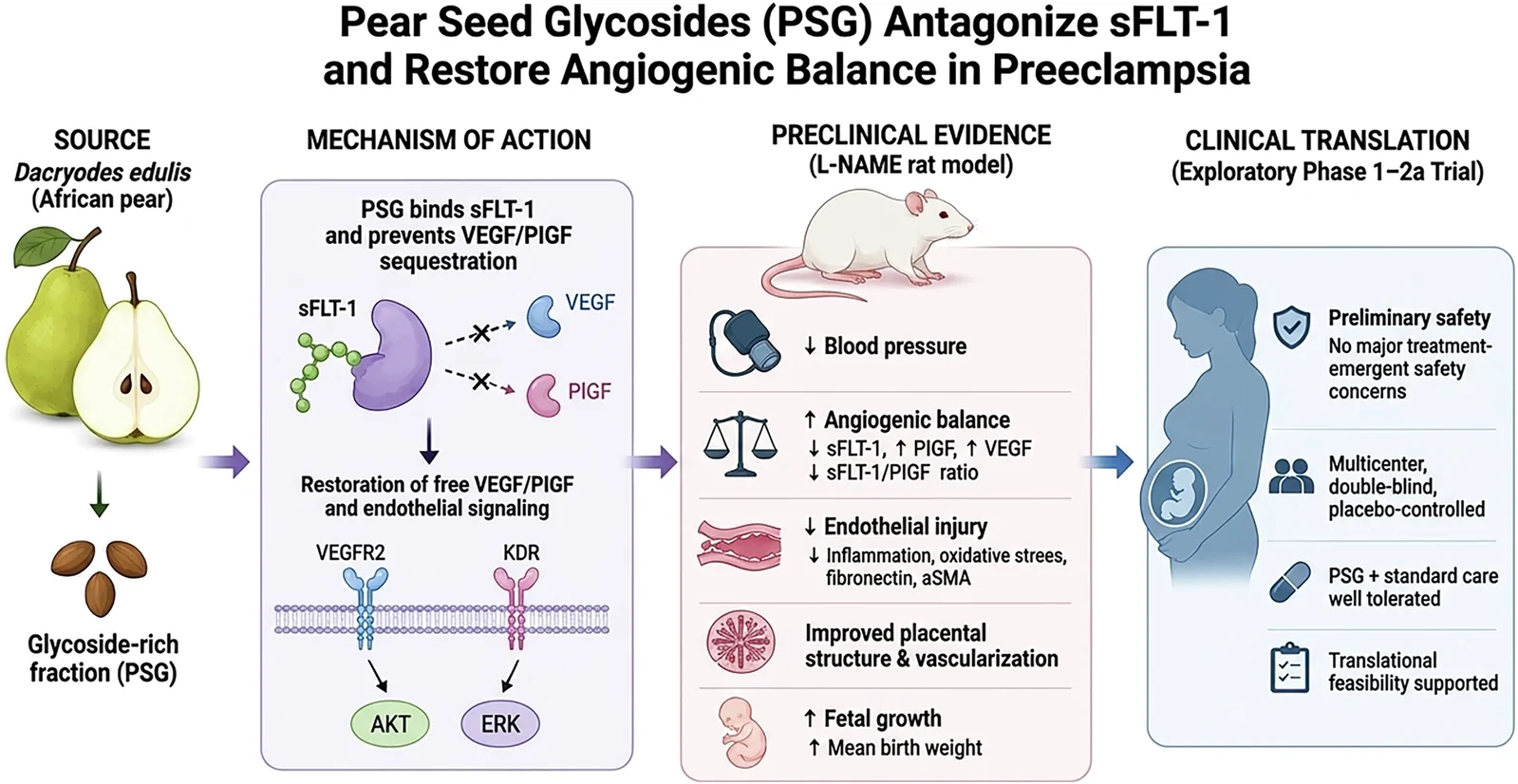

## Introduction

Preeclampsia is a multisystem hypertensive disorder of pregnancy and remains a major contributor to maternal and fetal morbidity and mortality worldwide [1, 2]. Despite advances in obstetric care, management remains largely supportive and focused on controlling maternal complications until delivery can be safely achieved. Current therapeutic approaches, including antihypertensive agents and magnesium sulfate, reduce maternal cardiovascular and neurological risk but do not directly reverse the endothelial and angiogenic disturbances that underlie disease progression [3]. Consequently, there remains considerable interest in identifying biologically targeted interventions capable of modulating the molecular pathways driving maternal vascular dysfunction in preeclampsia. A central feature of preeclampsia is the development of widespread endothelial dysfunction associated with placental stress and angiogenic imbalance [4, 5].

Among the mediators implicated in this process, soluble fms-like tyrosine kinase-1 (sFLT-1), a circulating soluble splice variant of VEGFR1, has emerged as a major pathogenic factor [6, 7]. Excess circulating sFLT-1 binds and sequesters vascular endothelial growth factor (VEGF) and placental growth factor (PlGF), thereby reducing pro-angiogenic signaling required for endothelial homeostasis [8, 9]. Elevated maternal sFLT-1 and reduced free PlGF are strongly associated with disease severity, vascular injury, placental insufficiency, and adverse maternal-fetal outcomes [10, 11]. This biology has positioned the sFLT-1/PlGF axis as both a biomarker system and a potential therapeutic target in preeclampsia. Several therapeutic strategies aimed at restoring angiogenic balance are currently under investigation [12]. These include extracorporeal approaches designed to reduce circulating sFLT-1 levels, RNA-based suppression of placental sFLT-1 production, and experimental interventions targeting downstream endothelial dysfunction [13, 14]. However, many of these approaches remain technically demanding, costly, or insufficiently characterized for widespread clinical implementation, particularly in resource-limited settings where the burden of preeclampsia is disproportionately high. Accordingly, there remains interest in identifying accessible and biologically active compounds capable of modulating angiogenic dysfunction through complementary mechanisms.

Natural-product-derived therapeutics have historically provided important leads for cardiovascular and inflammatory disease management, yet mechanistically defined interventions targeting angiogenic imbalance in preeclampsia remain limited. Pear seed preparations contain diverse bioactive constituents, including glycoside-enriched fractions with reported antioxidant, vascular, and anti-inflammatory properties [15]. However, their potential effects on endothelial dysfunction and angiogenic signaling in preeclampsia have not been systematically investigated. Importantly, most prior botanical studies in preeclampsia have remained descriptive, often lacking mechanistic interrogation of target engagement, endothelial signaling, or angiogenic ligand bioavailability [16–18].

In the present study, we combined endothelial, preclinical, biophysical, pharmacodynamic, and exploratory translational approaches to investigate the effects of pear seed glycosides (PSG) in preeclampsia-associated endothelial injury. We first examined whether pear seed extract modulates inflammatory and matrix-remodeling responses in TGFβ-stimulated endothelial cells and subsequently evaluated therapeutic effects in an L-NAME-induced rat model of preeclampsia. Bioactivity-guided fractionation was then used to identify glycosides as the principal active component of the extract. Mechanistically, we investigated whether PSG directly interacts with sFLT-1 and alters its sequestration of VEGF and PlGF using orthogonal biophysical and functional assays. Finally, we evaluated whether these mechanistic observations were associated with restoration of downstream angiogenic signaling and explored the safety and translational feasibility of PSG administration in participants with preeclampsia receiving standard clinical care. Collectively, these integrated findings provide a mechanistic and translational framework linking PSG-mediated endothelial protection, restoration of angiogenic bioavailability, functional antagonism of sFLT-1, and preliminary clinical feasibility in preeclampsia.

## Methodology

### In vitro studies

#### Purification of glycosides from the pear seeds

The procedure for the fractionation and purification was developed by the organic synthesis group of the Department of Chemistry, Imo State University, in collaboration with researchers at the Department of Chemistry, Monash University, Australia. Full details are described in the Supplementary File.

#### In vitro toxicity testing on epithelial, endothelial, stellate, and entero-endocrine cells

To evaluate the cytotoxicity and dose-dependent cellular responses to PSG, a panel of mammalian cell lines was exposed to varying concentrations of the extract (2–50 μg/mL). The cell lines included liver-derived HEPG2 and Huh7.5 cells, kidney epithelial cells (Qbi/HEK293), human umbilical vein endothelial cells (HUVECs), vascular smooth muscle cells (VSMCs), hepatic stellate cells (LX2), and enteroendocrine cells (STC-1). Cells were initially cultured in 10 cm dishes under standard conditions, then seeded onto 12-well plates and treated with PSG for 24 hours. Cell viability was assessed using the trypan blue exclusion assay to determine the percentage of viable versus non-viable cells following exposure [19].

#### Human umbilical vein endothelial cell culture

Cryopreserved human umbilical vein endothelial cells (HUVECs; Cell Applications Inc.), with fewer than two passages, were cultured in Gibco™ Human Large Vessel Endothelial Cell Basal Medium (LVEM) supplemented with Low Serum Growth Supplement (LSGS), containing 2% fetal bovine serum (v/v), 1 μg/mL hydrocortisone, 10 ng/mL human epidermal growth factor, and 3 ng/mL basic fibroblast growth factor. Cells were maintained at 37°C in a humidified incubator with 5% CO₂. Upon reaching 80% confluence, cells were seeded at a density of 3.5 × 10⁵ cells/mL in 6-well plates. Experimental treatments included 20 μg/mL PSG, 2.5 ng/mL TGFβ, or a combination of both. TGFβ was administered for 24 hours, while PSG was added 4 hours after TGFβ induction and maintained for the remaining 20 hours of incubation.

#### Transwell migration of HUVECs

HUVECs were harvested by trypsinization and seeded at a density of 5.0 × 10⁵ cells/mL into 8 μm pore-size Transwell® inserts placed within 12-well culture plates. To establish a chemotactic gradient, 500 μL of LVEM medium supplemented with LSGS and 20% fetal bovine serum was added to the lower chamber, ensuring contact with the underside of the insert. Following 24 hours of incubation at 37 °C, inserts were rinsed twice with 1× PBS and transferred to a new 12-well plate containing 1 mL of 70% ethanol for 10 minutes to fix the migrated cells. After fixation, the inserts were gently drained using sterile cotton-tipped applicators and air-dried for 15 minutes. Inserts were then stained with crystal violet for 10 minutes, washed in distilled water to remove excess stain, and allowed to dry completely. Migrated cells adhering to the underside of the membrane were imaged using an OMAX M837ZL-C140U3 microscope. Four representative fields per insert were captured and quantified. Relative cell migration was expressed as the average number of cells per field divided by the imaged area, as measured using OMAX ToupView software.

#### Immunoblotting

Following treatment, the culture medium was aspirated, and cells were washed twice with 1 mL of cold 1× PBS. Cells were harvested using a sterile cell scraper, resuspended in 1.5 mL PBS, and pelleted by centrifugation at 7000 rpm for 7 minutes at 4 °C. Cell pellets were lysed in buffer A (150 mM NaCl, 1% Triton X-100, 50 mM HEPES, pH 7.4) supplemented with 1× EDTA-free protease inhibitor cocktail (Roche). Lysis was performed on ice with intermittent vortexing every 10 minutes for a total of 30 minutes. Lysates were clarified by centrifugation at 14,000 rpm for 10 minutes at 4 °C, and the supernatants were collected for protein quantification using the Pierce™ BCA Protein Assay Kit (Thermo Fisher Scientific). Equal amounts of total protein were resolved by SDS-PAGE on a 10% polyacrylamide gel and transferred onto nitrocellulose membranes. Membranes were blocked with 0.5% skimmed milk in TBST for 1 hour at room temperature and incubated overnight at 4°C with primary antibodies against TNF-α (Proteintech, 17590-1-AP), α-Smooth Muscle Actin (α-SMA; Proteintech, 14395-1-AP), or Fibronectin (Proteintech, 15613-1-AP; 1:2500 dilution). The following day, membranes were washed and probed with DyLight™ 680-conjugated donkey anti-mouse IgG or DyLight™ 800-conjugated donkey anti-rabbit IgG secondary antibodies (Invitrogen) for 1 hour at room temperature. Fluorescent signals were visualized using the Odyssey Infrared Imaging System (LI-COR Biosciences), and band intensities were quantified using Odyssey software, with normalization to appropriate loading controls.

#### Quantitative real-time PCR

The RNeasy® Mini kit was used to extract total RNAs from the cultured HUVECs following the manufacturer’s instructions on the kit. cDNA synthesis was performed using the High-Capacity cDNA Reverse Transcription Kit, and following the kit’s protocol, the SYBR®Select Master Mix was applied to measure the triplicate determinations of the relative mRNA levels using the StepOnePlusTM qRT-PCR machine. Relative gene expression was normalized to the glyceraldehyde-3-phosphate dehydrogenase gene (GAPDH) and the primers for human mouse GAPDH, αSMA (ACTA2), and TNFα were synthesized by IDT from the PrimerQuest RT-PCR Design Tool. The human GAPDH primer sequence Forward - GGTGTGAACCATGAGAAGTATGA and Reverse- GAGTCCTTCCACGATACCAAAG, for ACTA2, forward - GATGGTGGGAATGGGACAAAA, Reverse - GCCATGTTCTATCGGGTACTTC, for TNFα, Forward - AGAGGGAGAGAAGCAACTACA, Reverse - GGGTCAGTATGTGAGAGGAAGA, and for fibronectin, Forward - CCACAGTGGAGTATGTGGTTA, Reverse - CAGTTCTTTAGGGCGATCAAT.

### In vivo experimental design

This study received ethical approval from the Imo State University ethics review board, under ethical approval number IMSU/BCM/ETS/20181219, and was conducted in compliance with the Principles of Laboratory Animal Care (NIH Publication No. 85-23). The sample size was calculated using the G*Power statistical software 3.1.9.7 with data from our pilot study measuring blood pressure. Standard deviation within each group (σ) was estimated at 5.945 mm/Hg and the effect size (f) was 0.827. For a α = β = 0.05 at a 95% significance level, a sample size of 21 experimental rats is needed.

Before commencing the efficacy study, pilot experiments were performed to optimize the route and timing of pear seed extract (PS) administration in the L-NAME-induced rat model of preeclampsia. To compare delivery routes, rats with established preeclampsia were randomized to receive PSG (40 mg/kg) by oral gavage, intravenous (IV), or intraperitoneal (IP) administration, while sham-treated animals received vehicle. Maternal systolic and diastolic blood pressure (SBP and DBP) and the circulating sFLT-1/PlGF ratio were measured at the study endpoint. To assess early pharmacodynamic responses, an independent cohort received a single PSG dose via the three administration routes, and SBP, DBP, and the sFLT-1/PlGF ratio were determined four hours later. Finally, to define the therapeutic window, PSG treatment was initiated either on gestational day 7 or day 16, and endpoint hemodynamic and angiogenic parameters were compared with untreated preeclamptic and normotensive pregnant controls.

#### Mating, induction of preeclampsia and grouping

For mating, 16-week-old inbred Wistar male rats (180–200 g) were housed overnight with non-pregnant females of similar age and weight, at a ratio of 1:3 (male:female), in clean, ventilated cages with commercial rodent feed (UAC Nigeria Vital Feeds, Grand Cereals, Jos, Nigeria) and water provided ad libitum. The estrous cycle was monitored via vaginal lavage, and pregnancy was confirmed the following morning by the presence of a copulatory plug or a positive vaginal smear [20]. This day was designated as gestational day 1. Pregnant females were then housed individually in metabolic cages and monitored throughout gestation. Preeclampsia was induced by daily intraperitoneal injection of N^G^-nitro-L-arginine methyl ester (L-NAME; 125 mg/kg) from gestational days 12 to 15, combined with 0.3 g/L L-NAME administered in the drinking water until the endpoint [21]. Preeclampsia was confirmed on day 15 based on elevated systolic/diastolic blood pressure and increased urinary protein concentration [21]. After confirmation, L-NAME injections were discontinued. Beginning on gestational day 16, preeclamptic rats received daily oral gavage of either PSG (200 mg/kg body weight) or fractionated PSG (50 mg/kg) at a volume of 1 mL, continued until euthanasia on gestational day 20. Details of the extraction and fractionation of PSG are provided in the Supplementary Materials. Seven normotensive pregnant rats receiving standard chow and saline served as controls. Fourteen rats with confirmed preeclampsia were randomly assigned to one of two groups: (i) placebo group (PE), receiving 1 mL of saline daily; and (ii) treatment group (PE + PS), receiving 1 mL of PSG extract (200 mg/mL). An additional group of five normotensive dams received PSG alone to assess potential adverse effects in healthy pregnancies.

#### Assessment of maternal characteristics

On gestational day 19, all pregnant dams were fasted for 12 hours overnight. Feed intake and maternal body weight were monitored daily from gestational day 15 to 20 using a precision automated balance. Diuresis was calculated as the average daily urine volume collected over the study period. Preliminary and endpoint urinary protein concentrations were measured on gestational days 15 and 20, respectively, using the Pierce™ BCA Protein Assay Kit (Thermo Fisher Scientific, Inc.). On gestational day 20, maternal systolic and diastolic blood pressure, as well as heart rate, were measured using a non-invasive tail-cuff system (CODA NIBP System, Kent Scientific Corporation, USA) in accordance with the manufacturer’s protocol. Fasting blood glucose and glycated hemoglobin (HbA1c) were measured via tail vein blood using an Accu-Chek glucometer and a Siemens/Bayer DCA 2000+ Analyzer, respectively. Following cardiovascular assessments, animals were anesthetized with intraperitoneal pentobarbital sodium (3%, 50 mg/kg). A midline abdominal incision was performed, and maternal blood (3 mL) was collected via cardiac puncture using a sterile 5 mL syringe and transferred into heparinized tubes. The uterine horns were exposed, excised, rinsed in Dulbecco’s phosphate-buffered saline (PBS), and weighed. Under a binocular dissecting microscope, uterine arteries and placentas were isolated. Each placenta and uterine horn was weighed using a sensitive digital scale. The maternal heart and placentas were subsequently fixed in 10% neutral-buffered formalin, paraffin-embedded, sectioned, and stained with hematoxylin and eosin (H&E) for histological evaluation. Plasma was prepared by centrifuging whole blood at 2500 × *g* for 10 minutes, followed by storage at −20°C until analysis. Plasma insulin concentrations were determined using a rat-specific Ins1/Insulin ELISA kit, following the manufacturer’s instructions.

The Homeostatic Model of Assessment for Insulin Resistance (HOMA-IR) was calculated according to the formula:

$${{{\rm{HOMA}}}}-{{{\rm{IR}}}}=[{{{\rm{glucose}}}}({{{\rm{mmol}}}}/{{{\rm{L}}}})* {{{\rm{insulin}}}}({{{\rm{\mu iU}}}}/{{{\rm{mL}}}})/22.5],$$

where 1 mg/dl equals 0.055 mmol/l.

The glomerular filtration rate (GFR) estimation was obtained from the rate of creatinine clearance according to the online calculator of the National Kidney Foundation. No adjustments were made to the surface area of the dams. Anion gap was estimated by subtracting serum anions (Cl^-^ and HCO_3_^-^) from the serum cations (Na^+^ and K^+^) while the levels of the serum electrolytes were determined using an Audicom (Jiangsu, China) automated electrolyte analyzer, and creatinine levels were measured using an Abcam creatinine colorimetric assay kit. Cardiac risk ratio (CRR) was calculated from the ratio of total cholesterol to high-density lipoprotein that were measured using a BT-3000 auto analyzer (Biotecnica Instruments, Italy).

#### Clinical chemistry

Creatine kinase was assayed by spectrophotometry (Sigma Aldrich Creatine Kinase Activity Assay Kit MAK116), lactate dehydrogenase by enzymatic colorimetry using the methods described in the Lactate Dehydrogenase Kit (Sigma Aldrich St. Louis, MO, USA, Catalog No-MAK066) while the cardiac troponin and angiotensin II assay was performed by enzyme-linked immunosorbent assay (ELISA) with the Rat Cardiac Troponin T (cTn-T) and Rat angiotensin II (ANGII) ELISA kit respectively (Cusabio Technology, Houston, TX, USA). Plasma and placental TNFα, and TNFR1 were measured with a commercial ELISA kit (R&D Systems); Rat TNF-alpha Quantikine ELISA Kit and Mouse/Rat TNF RI/TNFRSF1A Quantikine ELISA Kit while IL-1 β, IL-6, and IFN-γ were quantified with Rat IL-1 β (RAB0278), Rat IL-6 (RAB0311), and Rat IFNγ (RAB0227) ELISA Kit (Sigma Aldrich, USA) respectively.

Serum levels of malondialdehyde were measured as described by Ohkawa et al., through the thiobarbituric acid reactive substances (TBARS) assay [22]. The superoxide dismutase (SOD) serum levels were determined through the inhibition of pyrogallol autoxidation [23]. Catalase was measured as the rate of decomposition of H_2_O_2_ at 240 nm for 30 seconds intervals for 5 min following the method described [24]. The plasma levels of sFlt-1, PlGF, and VEGF were measured with commercial ELISA kits VEGFR1/FLT-1 (RAB1934), PLGF-2 (RAB0404), VEGF ELISA Kit (RAB0511) from Sigma-Aldrich USA.

#### Uterine artery stimulation

Uterine arteries of rats from the respective groups were isolated and incubated in estradiol or angiotensin II. Precisely 2.5 mm length of the main uterine artery, cut between the middle and lower third of the vascular arcade, was obtained and placed for 6 hours in physiological salt solution (PSS) containing a range of concentrations, 1e^-11^M - 5e^-5^M, of either β-Estradiol (E2758) or angiotensin II (05-23-0101) from Sigma-Aldrich, USA. After incubation, the artery was homogenized and assayed for both angiotensin II type I receptor (AT1R) with the commercial ELISA kit, Angiotensin II Receptor 1 ELISA Kit (Biomatik Inc., Canada), and total ATPase according to our earlier described procedure [20].

##### Endothelial module rescue and angiogenic signaling analyses

*HUVEC culture and treatment conditions*: Primary human umbilical vein endothelial cells (HUVECs) were maintained in endothelial growth medium supplemented with endothelial growth supplements and 10% fetal bovine serum under standard humidified culture conditions (37 °C, 5% CO₂). Cells between passages 3-6 were used for all experiments. For endothelial injury studies, HUVECs were serum-reduced overnight before stimulation with recombinant human sFLT-1 in the presence or absence of pear seed glycosides (PSG) at the indicated concentrations (10-50 μg/mL). Vehicle-treated cells served as controls. For signaling rescue experiments, cells were additionally stimulated with recombinant human PlGF prior to endpoint collection.

*Gene-expression and endothelial module analyses*: Total RNA was isolated using TRIzol reagent followed by reverse transcription using a cDNA synthesis kit according to the manufacturer’s instructions. Quantitative real-time PCR (qRT-PCR) was performed using SYBR Green chemistry on a real-time PCR platform. Relative gene expression was normalized to GAPDH using the 2^-ΔΔCt method. Endothelial injury modules were constructed a priori based on biologically related gene sets representing angiogenic bioavailability (VEGFA, PGF, KDR, NRP1, NOS3), EndMT/fibronectin remodeling (ACTA2, VIM, SNAI1, FN1, COL1A1), and inflammatory endothelial activation (TNF, IL6, ICAM1, VCAM1). Relative module scores were calculated using normalized mean-centered expression values across genes within each module.

*Immunoassays for signaling pathway activation*: Phosphorylated VEGFR2/KDR, AKT S473, and ERK1/2 signaling were quantified using commercially available ELISA kits according to the manufacturer’s instructions. Total ERK measurements were used for normalization of pERK/ERK ratios where indicated. Absorbance was measured using a microplate reader, and concentrations were normalized to total protein content. All experiments were independently repeated at least three times.

##### Biophysical characterization of PSG-sFLT-1 interactions

*Preparation of PSG fractions:* Pear seed glycoside preparations were generated through sequential fractionation of crude pear seed extract. Active glycoside-enriched fractions (including fraction F3) were isolated using solvent partitioning and chromatographic enrichment procedures. Heat-inactivated PSG preparations were generated by thermal denaturation prior to use. Depleted PSG fractions were prepared by removal of glycoside-enriched components during fractionation. Vehicle-only preparations served as negative controls.

*Surface plasmon resonance (SPR) / bio-layer interferometry (BLI) binding analyses:* Direct PSG-sFLT-1 interactions were evaluated using SPR/BLI-based binding assays. Recombinant human sFLT-1 protein was immobilized onto sensor surfaces according to the manufacturer’s recommendations. PSG preparations, control fractions, VEGF-A, and PlGF were introduced at increasing concentrations under continuous flow conditions. Association and dissociation responses were monitored in real time. Apparent equilibrium binding parameters and relative interaction strengths were derived from dose-response curves after subtraction of vehicle-control signals.

*Microscale thermophoresis (MST):* Solution-phase PSG-sFLT-1 interactions were independently validated using microscale thermophoresis (MST). Fluorescently labeled recombinant human sFLT-1 protein was incubated with serial dilutions of PSG preparations prior to thermophoretic analysis. Relative fluorescence changes (ΔFnorm) were quantified across PSG concentrations to estimate apparent interaction behavior independently of surface immobilization.

*Binding specificity and concordance analyses:* Specificity analyses were performed using heat-inactivated PSG, depleted PSG fractions, vehicle controls, and unrelated recombinant proteins. Positive-control ligand interactions with VEGF-A and PlGF were included for comparison. Cross-platform concordance between SPR/BLI- and MST-derived interaction profiles was assessed by correlation analysis using independently derived apparent affinity estimates.

##### Functional antagonism of sFLT-1-mediated ligand sequestration

*Competitive ligand-binding ELISA:* Competitive ELISA assays were performed to determine whether PSG interferes with sFLT-1-mediated binding of PlGF and VEGF-A. Recombinant human sFLT-1 was incubated with increasing concentrations of PSG prior to exposure to recombinant PlGF or VEGF-A. Bound ligand complexes were quantified using enzyme-linked detection systems according to the manufacturer’s instructions. Relative inhibition of ligand sequestration was calculated relative to sFLT-1-only controls.

*BLI competition assays:* Functional competition analyses were additionally performed using BLI-based interaction assays. Recombinant sFLT-1-coated biosensors were exposed to PlGF or VEGF-A in the presence of increasing PSG concentrations. Reductions in association signal relative to untreated controls were interpreted as PSG-mediated disruption of ligand sequestration.

*Free and bound PlGF redistribution analyses:* To assess redistribution of angiogenic ligand pools, concentrations of free PlGF and sFLT-1-bound PlGF complexes were quantified following PSG treatment. Recombinant binding systems were incubated with increasing PSG concentrations prior to measurement of free and bound ligand species using ELISA-based detection methods. Relative redistribution profiles were calculated across PSG concentrations.

*Ex vivo PlGF bioavailability assays*: Ex vivo bioavailability studies were performed by incubating PSG with pre-formed sFLT-1/PlGF complexes over time. Free bioavailable PlGF was quantified at the indicated time points using ELISA-based detection assays.

### Exploratory Clinical trial

#### Safety and dose-escalation trial of pear seed glycosides (PSG)

This study was conducted as a multicenter, double-blind, randomized phase 1-2a clinical trial to evaluate the safety, tolerability, and preliminary efficacy of pear seed glycosides (PSG) in participants with preeclampsia. Participants were recruited across five affiliated clinical sites within the Imo State University (IMSU) clinical trial network, each maintaining participant retention rates exceeding 80%. Ethical approval was obtained from the IMSU Ethics Review Board (Ref: IMSU/ETS/BCH26), and the trial was registered with the WHO International Clinical Trials Registry Platform (PACTR202405559013998). Eligible participants were randomized to receive PSG or placebo in addition to standard clinical care. PSG administration and participant monitoring were performed by designated clinicians or trial coordinators at each participating site. Investigators, clinical personnel, outcome assessors, and study contributors remained blinded to treatment allocation throughout the study period. Trial oversight, safety monitoring, and inter-site coordination were conducted by the Clinical Trial Steering Group (CTSG). Detailed study protocols, clinical procedures, and safety monitoring frameworks are provided in the Supplementary Materials.

#### Inclusion and exclusion criteria

Demographic characteristics, medical history, and obstetric information were collected at enrollment using standardized clinical questionnaires and case-report forms across all participating sites. All participants continued to receive standard medical management for preeclampsia as determined by their attending physicians throughout the study period. Eligible participants were pregnant women between 20 and 25 weeks of gestation with an antepartum diagnosis of preeclampsia, defined by systolic and/or diastolic blood pressure ≥140/90 mmHg together with a spot urine protein-to-creatinine ratio >30 mg/mmol confirmed on two independent assessments during the early third trimester. Enrollment required fulfillment of all diagnostic criteria, provision of written informed consent, and willingness to receive the assigned study intervention. Exclusion criteria included gestational age >27 weeks at enrollment; obesity; gestational diabetes mellitus; chronic hypertension; malignancy; thyroid, adrenal, renal, or hepatic disorders; and hematologic disorders other than mild iron-deficiency anemia. Pregnancies complicated by known chromosomal abnormalities, major congenital malformations, fetal demise, or planned termination were also excluded. Additional exclusion criteria included prior antihypertensive or diuretic use before the third trimester and concurrent participation in another interventional clinical trial. Detailed inclusion and exclusion criteria are provided in the Supplementary Materials.

#### Dose estimation for participants with preeclampsia

The starting human-equivalent dose of pear seed glycosides (PSG) was estimated from the no observed adverse effect level (NOAEL) identified in preclinical rat studies using standard FDA-recommended allometric scaling for interspecies dose conversion. The NOAEL of 50 mg/kg in rats corresponded to an estimated human-equivalent starting dose of 7.6 mg/kg body weight. Initial safety and dose-escalation assessments were informed by a preceding evaluation in normotensive pregnant women (PACTR202206531545729). For the present phase 1-2a trial, pregnant women between 20 and 25 weeks of gestation were screened for eligibility, followed by randomization between 28 and 31 weeks of gestation after completion of a three-week run-in phase. Among 125 screened participants, 83 met eligibility criteria and were randomized to PSG or placebo treatment groups. Across participating trial sites, approximately 80% of enrolled participants completed the  intervention. PSG administration was initiated at 7 mg/kg body weight twice weekly and progressively escalated to 20, 30, 40, and 50 mg/kg to evaluate tolerability and maternal-fetal safety. Transient maternal hypotension and evidence of fetal distress were observed at 50 mg/kg, leading to selection of 40 mg/kg as the maximum tolerated dose for continued administration through delivery. During the initial two weeks of intervention, participants underwent intensive in-person monitoring by dedicated study nurses, including serial assessment of maternal blood pressure and fetal heart rate. Adverse events were documented according to prespecified trial safety protocols. Subsequent monitoring was performed during routine antenatal visits by site coordinators and trial hub managers. Maternal and fetal clinical parameters were monitored longitudinally until delivery. Peripheral blood and urine samples were collected at baseline and during follow-up visits, while placental tissue and cord blood were obtained within one hour of delivery. All biospecimens were processed and stored under standardized site-specific conditions before transfer to the central laboratory for downstream analyses.

#### Outcome measurements

Maternal safety outcomes were prospectively assessed throughout the study period and included respiratory, gastrointestinal, neurological, cardiovascular, renal, and systemic adverse events, including cough, wheezing, diarrhea, chloasma, headache, heartburn, swelling, nausea, constipation, fatigue, fever, seizure activity, oliguria, pulmonary edema, myocardial infarction, stroke, dialysis requirement, heart failure, and end-stage organ dysfunction. Fetal and neonatal safety outcomes included fetal growth restriction, placental abruption, fetal distress, perinatal mortality, congenital malformations, low birth weight, and neonatal intensive care unit (NICU) admission. Maternal systolic and diastolic blood pressure measurements were recorded longitudinally throughout the intervention period, and urine protein levels were assessed at scheduled antenatal visits to evaluate changes in disease severity following PSG administration at the selected tolerated dose of 40 mg/kg relative to placebo-treated participants.

### Statistical analysis

Statistical analyses were performed using GraphPad Prism and SPSS. Data are presented as mean ± SEM unless otherwise stated. Normality was assessed using the Shapiro-Wilk test. Comparisons between two groups were performed using unpaired two-tailed Student’s t-tests or Mann-Whitney U tests where appropriate. Multiple-group comparisons were analyzed using one-way or two-way ANOVA followed by Tukey’s multiple-comparison correction. Repeated-measures analyses were applied for uterine artery dose-response experiments. For qRT-PCR studies, relative gene expression was calculated using the 2^-ΔΔCt method following normalization to GAPDH. Endothelial module scores were generated from predefined angiogenic, inflammatory, and EndMT-associated gene sets using normalized mean-centered expression values. SPR/BLI, MST, competitive ELISA, and ligand sequestration assays were analyzed using nonlinear regression and concentration-response modeling to derive apparent binding and inhibitory parameters. Cross-platform concordance analyses were performed using correlation-based methods. Clinical trial analyses were conducted primarily as exploratory safety and tolerability assessments. Continuous variables were compared using parametric or non-parametric tests as appropriate, while categorical adverse-event frequencies were analyzed using Fisher’s exact or chi-square tests. No formal multiplicity correction was applied to exploratory clinical endpoints. Two-sided P values < 0.05 were considered statistically significant.

## Results

### Pear seed extract suppresses endothelial inflammatory and matrix-remodeling responses in TGFβ-stimulated HUVECs

**Fig. 1:**
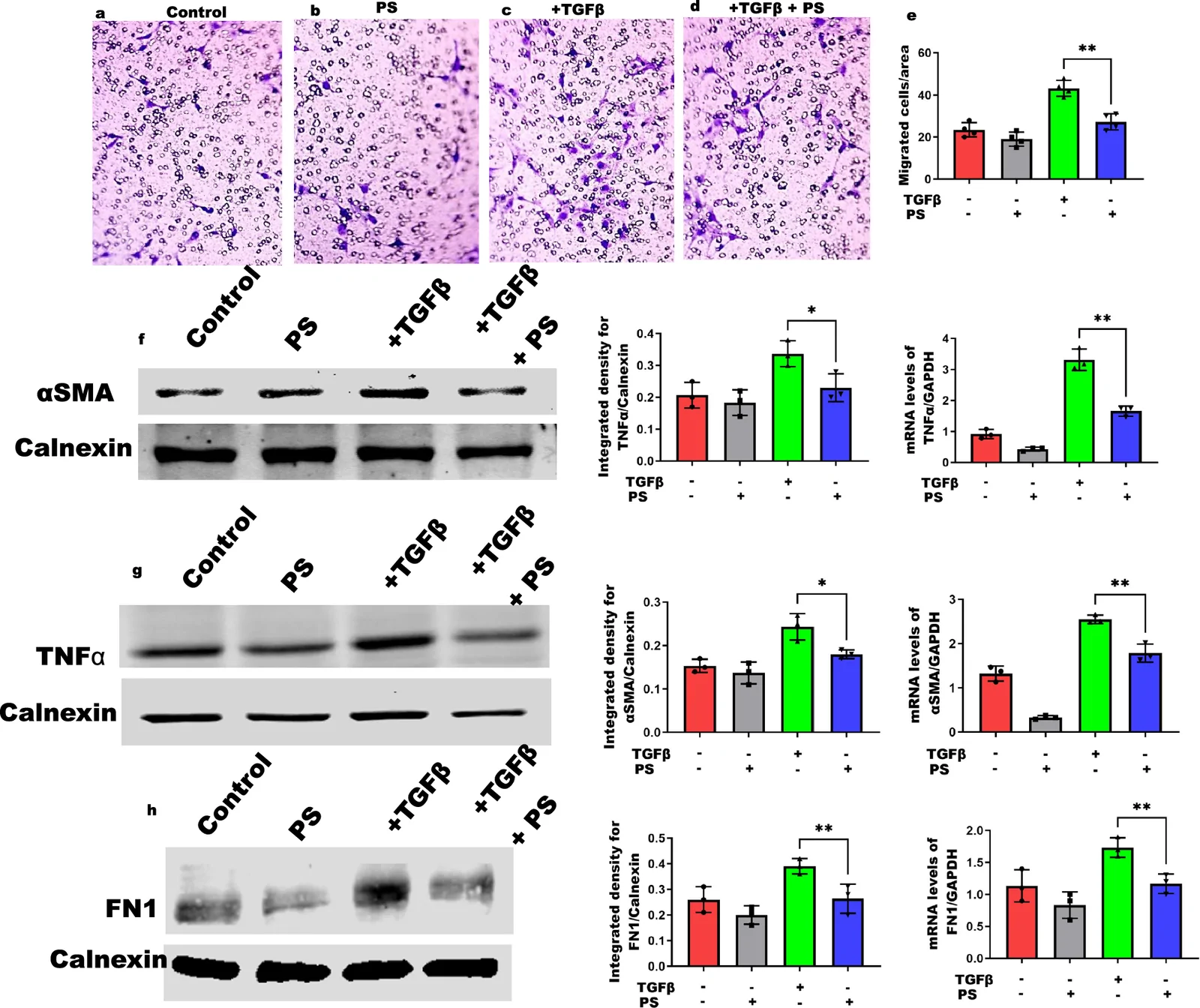
Effect of pear seed extract on TGFβ-treated HUVECs. Representative transwell migration images of HUVECs treated with control (**a**), pear seed extract (PS; **b**), TGFβ (**c**), or TGFβ plus PS (**d**). Quantification of migrated cells is shown in (**e**). Immunoblot and corresponding mRNA analyses of α-smooth muscle actin (αSMA; **f**), tumor necrosis factor-α (TNFα; **g**), and fibronectin (FN1; **h**) in HUVECs under the indicated treatment conditions. Calnexin was used as the loading control for immunoblot analyses, and GAPDH was used for normalization of mRNA expression

To determine whether pear seed extract (PS) modulates endothelial injury-associated responses, HUVECs were exposed to TGFβ in the presence or absence of PS. Representative transwell analyses demonstrated that TGFβ increased endothelial migration relative to untreated controls (Fig. 1a–e), whereas co-treatment with PS reduced the number of migrated cells. Immunoblot and matched transcript analyses further showed increased expression of α-smooth muscle actin (αSMA), tumor necrosis factor-α (TNFα), and fibronectin (FN1) following TGFβ stimulation (Fig. 1f–h). PS co-treatment attenuated the induction of these markers at both protein and mRNA levels. Consistent with these findings, extracellular fibronectin accumulation was increased in TGFβ-treated HUVECs and reduced in cells co-treated with PS (Fig. 1i–k). Together, these data show that PS modulates migratory, inflammatory, and extracellular matrix-associated responses in TGFβ-stimulated endothelial cells.

### Pear seed extract attenuates cardiovascular, inflammatory, and angiogenic features of L-NAME-induced preeclampsia

**Fig. 2:**
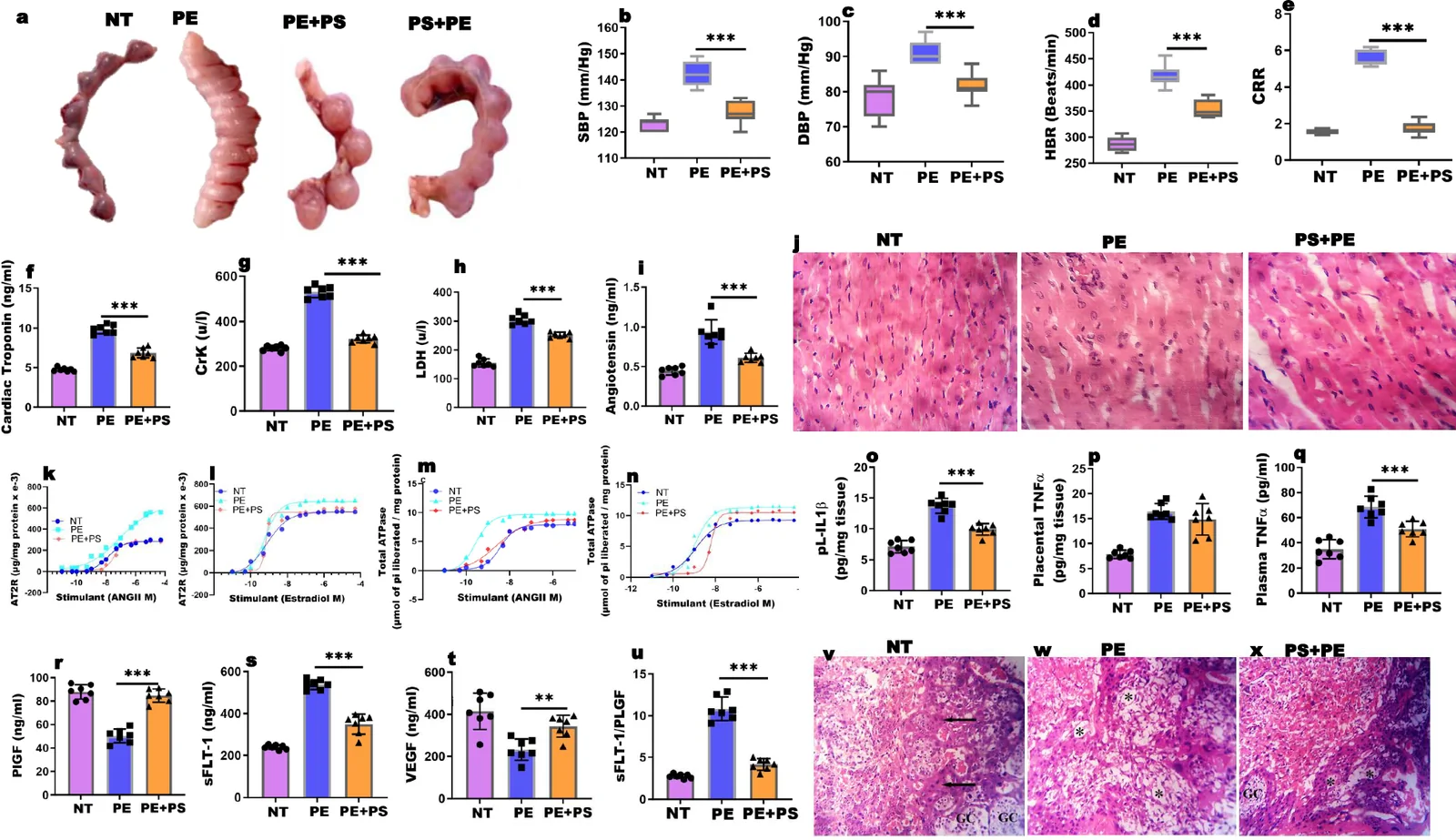
Effect of pear seed extract in an L-NAME-induced rat model of preeclampsia. Representative uterine horns from normotensive (NT), preeclamptic (PE), PE treated with pear seed extract (PE + PS), and PS pre-treatment groups are shown in (**a**). Systolic blood pressure (SBP; **b**) and diastolic blood pressure (DBP; **c**) were measured in NT, PE, and PE + PS rats. Heart rate (HR; **d**) and cardiac risk ratio (CRR; **e**) are shown for the indicated groups. Cardiac troponin (**f**), creatine kinase (CK; **g**), lactate dehydrogenase (LDH; **h**), and angiotensin levels (**i**) were quantified in NT, PE, and PE + PS rats. Representative histological sections of cardiac tissue are shown in (**j**). Dose-response curves of uterine arteries following angiotensin II- (**k**,** m**) or estradiol-mediated (**l**, **n**) ex vivo stimulation is shown for the indicated groups. Placental interleukin-1β (pL-IL1β; **o**), placental TNFα (**p**), and plasma TNFα (**q**) were measured in NT, PE, and PE + PS rats. Placental growth factor (PlGF; **r**), soluble fms-like tyrosine kinase-1 (sFLT-1; **s**), vascular endothelial growth factor (VEGF; **t**), and the sFLT-1/PlGF ratio (**u**) were quantified in the indicated groups. Representative placental histological sections from NT (**v**), PE (**w**), and PE + PS (**x**) rats are shown

Having established that pear seed extract (PS) suppresses endothelial migratory, inflammatory, and fibronectin-remodeling responses in TGFβ-stimulated HUVECs (Fig. 1), and after optimizing the route and timing of administration in pilot studies (Supplementary Figs. 1–3) we next evaluated whether these endothelial-protective effects translated into therapeutic benefit in vivo using an L-NAME-induced rat model of preeclampsia. Pilot studies demonstrated comparable efficacy following different routes of administration, supporting selection of oral dosing for the definitive efficacy study (Supplementary Fig. 1). Gross examination revealed marked abnormalities in uterine morphology in preeclamptic animals, whereas PS-treated rats exhibited improved uterine appearance (Fig. 2a). L-NAME administration induced significant elevations in systolic and diastolic blood pressure, heart rate, and cardiac risk ratio, all of which were attenuated following PS treatment (Fig. 2b, e). Consistent with reduced cardiovascular injury, circulating cardiac troponin, creatine kinase, lactate dehydrogenase, and angiotensin levels were also reduced in PS-treated animals relative to untreated preeclamptic rats (Fig. 2f–i). Histological assessment further demonstrated structural alterations in cardiac and placental tissues in preeclamptic animals that were less pronounced following PS administration (Fig. 2j, v–x). PS additionally modulated uterine vascular responsiveness, inflammatory cytokine levels, and angiogenic balance, including restoration of PlGF and VEGF together with reduction of sFLT-1 and the sFLT-1/PlGF ratio (Fig. 2k–u).

Consistent with these findings, pilot studies demonstrated that PS remained effective when treatment was initiated after establishment of the preeclamptic phenotype, although earlier administration attenuated disease severity without completely preventing disease onset (Supplementary Figure 2).

### Bioactivity-guided fractionation prioritizes glycosides as the principal anti-preeclamptic component of pear seed extract

**Fig. 3:**
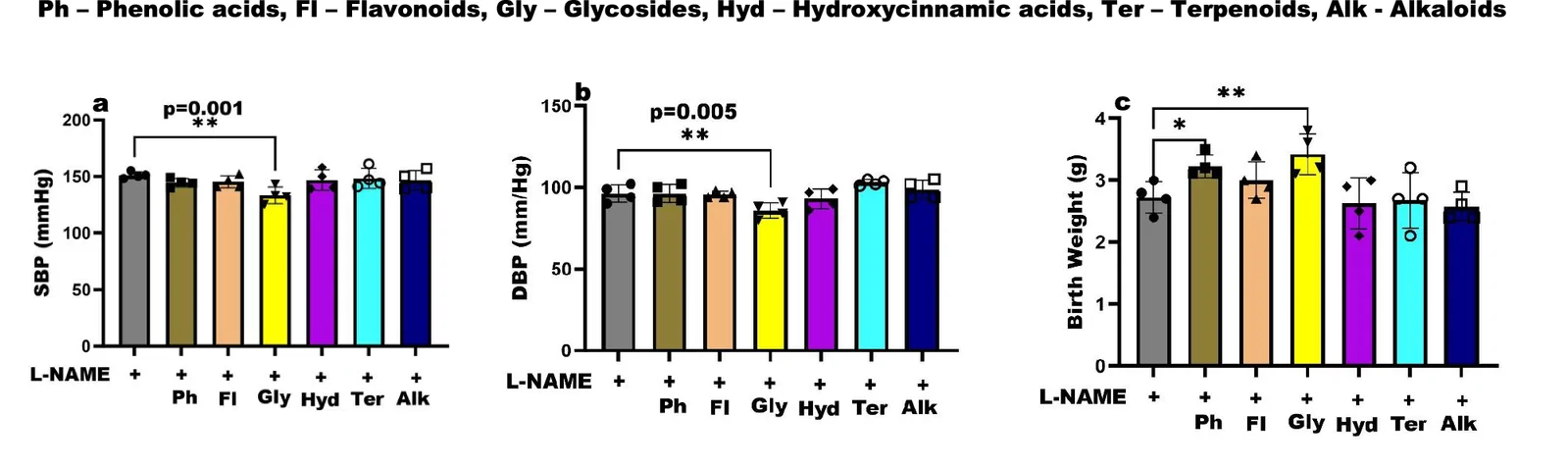
Effect of pear seed extract fractions on maternal blood pressure and fetal growth in L-NAME-induced preeclampsia. Systolic blood pressure (SBP; **a**), diastolic blood pressure (DBP; **b**), and fetal birth weight (**c**) were measured in L-NAME-treated rats receiving different fractions of pear seed extract. Fractions analyzed included phenolic acids (Ph), flavonoids (Fl), glycosides (Gly), hydroxycinnamic acids (Hyd), terpenoids (Ter), and alkaloids (Alk). Data are presented as mean ± SEM. *P < 0.05, **P < 0.01

Having demonstrated that pear seed extract (PS) improves endothelial dysfunction and preeclampsia-associated vascular injury, we next performed bioactivity-guided fractionation to identify the principal therapeutic component within the extract. Among the fractions tested, the glycoside-enriched fraction produced the greatest reduction in systolic and diastolic blood pressure in L-NAME-treated rats (Fig. 3a,b). In parallel, the glycoside fraction also showed the greatest improvement in fetal birth weight relative to untreated preeclamptic animals (Fig. 3c). Other fractions, including phenolic acids, flavonoids, hydroxycinnamic acids, terpenoids, and alkaloids, demonstrated comparatively weaker effects across these parameters.

### PSG restores angiogenic signaling and suppresses sFLT-1-driven endothelial injury programs

**Fig. 4:**
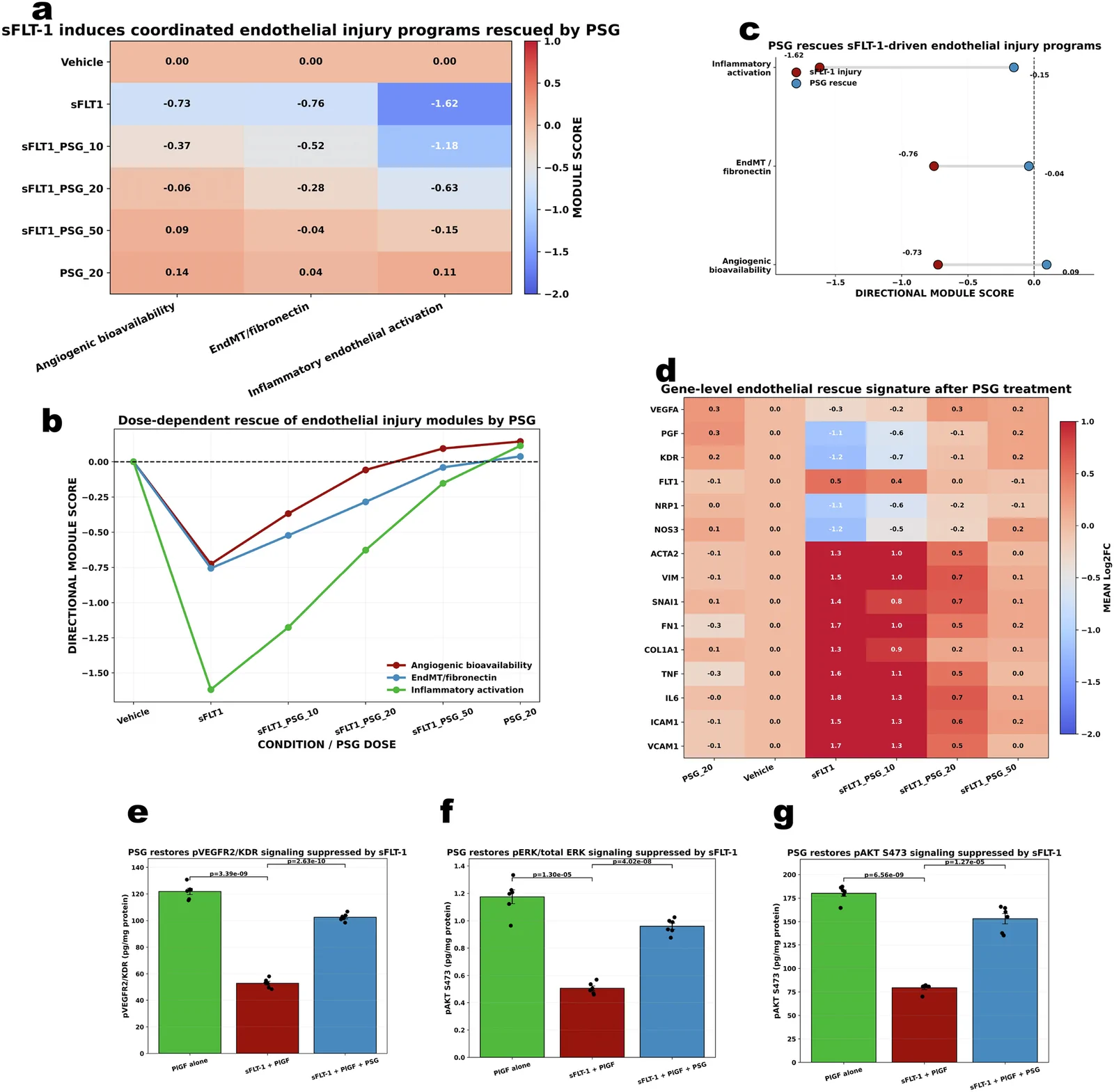
PSG restores angiogenic signaling and rescues sFLT-1-induced endothelial injury programs in HUVECs. Integrated module score heatmap showing angiogenic bioavailability, EndMT/fibronectin, and inflammatory endothelial activation signatures in vehicle-treated, sFLT-1-treated, and PSG-treated HUVECs is shown in (**a**). Module prioritization analysis comparing sFLT-1-induced endothelial injury and PSG-mediated rescue across signaling modules is shown in (**b**). Dose-dependent rescue of angiogenic bioavailability, EndMT/fibronectin, and inflammatory activation module scores following PSG treatment is shown in (**c**). Gene-level heatmap showing relative expression changes of angiogenic, inflammatory, and EndMT-associated genes across treatment conditions is shown in (**d**). Quantification of pVEGFR2/KDR (**e**), pERK/total ERK (**f**), and pAKT S473 (**g**) signaling in HUVECs treated with PlGF alone, sFLT-1 plus PlGF, or sFLT-1 plus PlGF and PSG. Color scales represent normalized module scores or mean log2 fold-change values. Data are presented as mean ± SEM

Having identified glycosides as the principal bioactive component of pear seed extract (Fig. 3), in addition to the independent pharmacodynamic study showing that improvement in the circulating sFLT-1/PlGF ratio occurred within four hours of PSG administration (Supplementary Fig. 3), we next investigated whether PSG directly modulates endothelial injury programs induced by recombinant sFLT-1. Integrated module-score analysis demonstrated that sFLT-1 exposure suppressed angiogenic bioavailability signatures while simultaneously increasing EndMT/fibronectin and inflammatory endothelial activation programs in HUVECs (Fig. 4a). PSG treatment progressively reversed these transcriptional changes across all three modules. Comparative module prioritization analysis further demonstrated marked perturbation of inflammatory and EndMT-associated pathways following sFLT-1 exposure, with substantial normalization following PSG treatment (Fig. 4b). Dose-response analyses showed progressive restoration of angiogenic signaling together with attenuation of inflammatory and fibronectin-remodeling signatures as PSG concentration increased (Fig. 4c). At the gene level, sFLT-1 reduced expression of angiogenic regulators including VEGFA, PGF, KDR, NRP1, and NOS3 while increasing expression of inflammatory and mesenchymal markers including TNF, IL6, ICAM1, VCAM1, ACTA2, VIM, SNAI1, FN1, and COL1A1 (Fig. 4d). Consistent with these transcriptional changes, sFLT-1 suppressed VEGFR2/KDR, ERK, and AKT signaling despite PlGF stimulation, whereas PSG restored phosphorylation of these downstream signaling mediators (Fig. 4e–g).

### PSG directly binds and functionally engages sFLT-1 through orthogonal biophysical assays

**Fig. 5:**
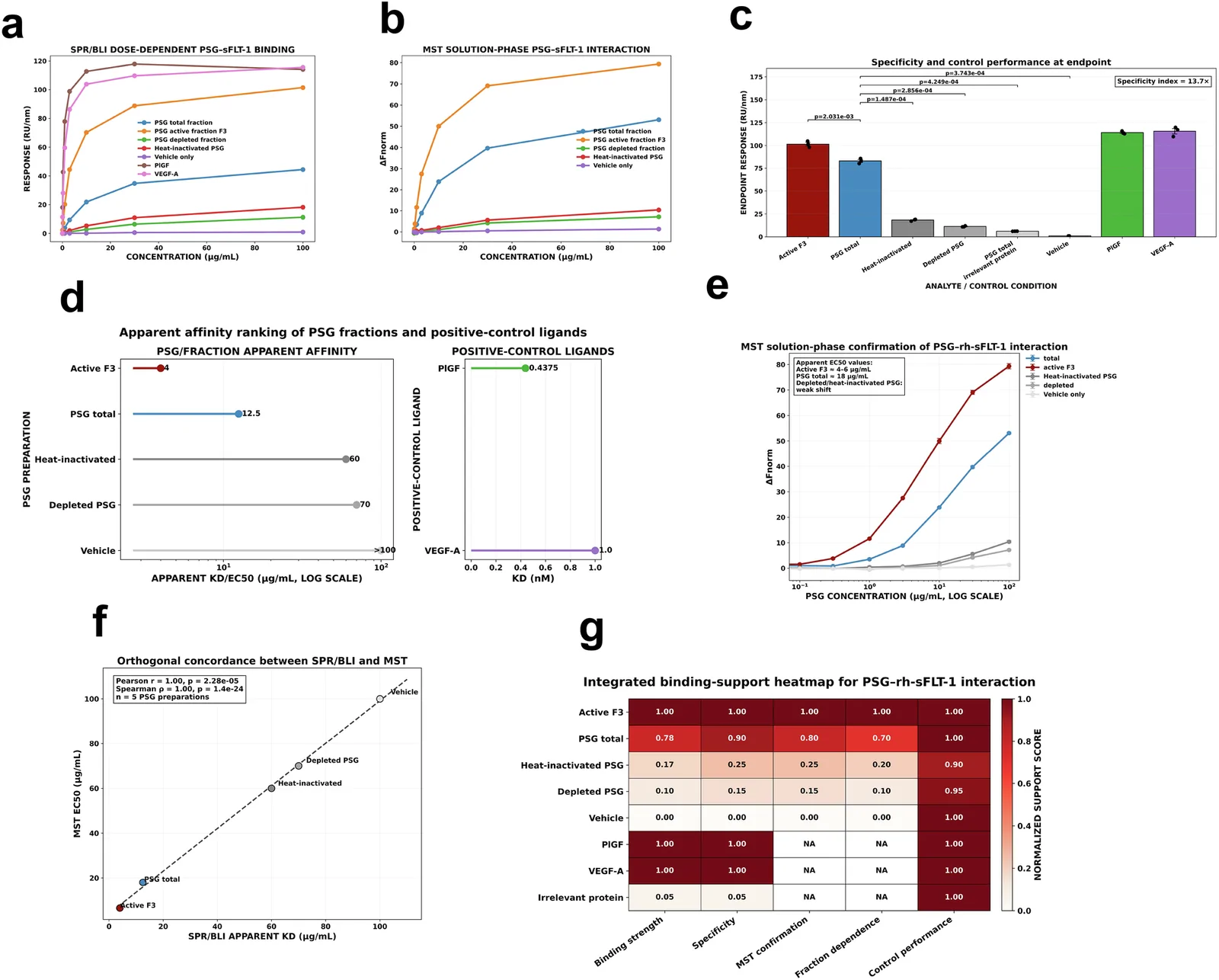
Biophysical characterization of PSG-sFLT-1 interactions. SPR/BLI dose-response analysis of PSG total extract, PSG active fraction F3, heat-inactivated PSG, depleted PSG, vehicle control, PlGF, and VEGF-A interactions with recombinant human sFLT-1 is shown in (**a**). MST solution-phase interaction analysis of PSG preparations with recombinant human sFLT-1 is shown in (**b**). Endpoint specificity and control-performance analysis comparing PSG preparations, depleted fractions, vehicle controls, and positive-control ligands is shown in (**c**). Apparent affinity ranking of PSG fractions and positive-control ligands derived from binding analyses is shown in (**d**). Independent MST solution-phase confirmation of PSG-rh-sFLT-1 interactions across PSG concentrations is shown in (**e**). Orthogonal concordance analysis comparing apparent affinities derived from SPR/BLI and MST platforms is shown in (**f**). Integrated binding-support heatmap summarizing binding strength, specificity, MST confirmation, fraction dependence, and control performance across PSG preparations and controls is shown in (**g**). Color scales represent normalized support scores

Following identification of PSG as the principal bioactive fraction of pear seed extract (Fig. 3), we next investigated whether PSG directly interacts with soluble fms-like tyrosine kinase-1 (sFLT-1). SPR/BLI analyses demonstrated concentration-dependent interactions between recombinant human sFLT-1 and PSG preparations, with the active F3 glycoside fraction exhibiting the strongest binding response relative to total PSG, heat-inactivated PSG, depleted fractions, and vehicle controls (Fig. 5a). Consistent findings were obtained using MST-based solution-phase interaction analyses, confirming PSG-sFLT-1 engagement independently of surface immobilization conditions (Fig. 5b, e). Endpoint specificity analyses further demonstrated substantially reduced interaction signals in depleted, heat-inactivated, and vehicle-treated controls relative to active PSG fractions and positive-control ligands (Fig. 5c). Apparent affinity ranking identified the active glycoside fraction as the strongest PSG-derived interacting preparation (Fig. 5d). Comparative analyses across SPR/BLI and MST platforms demonstrated strong concordance between independently derived interaction parameters (Fig. 5f). Integrated scoring across orthogonal assays further supported selective enrichment of sFLT-1 interaction activity within PSG glycoside fractions (Fig. 5g).

### PSG functionally antagonizes sFLT-1-mediated sequestration of PlGF and VEGF

**Fig. 6:**
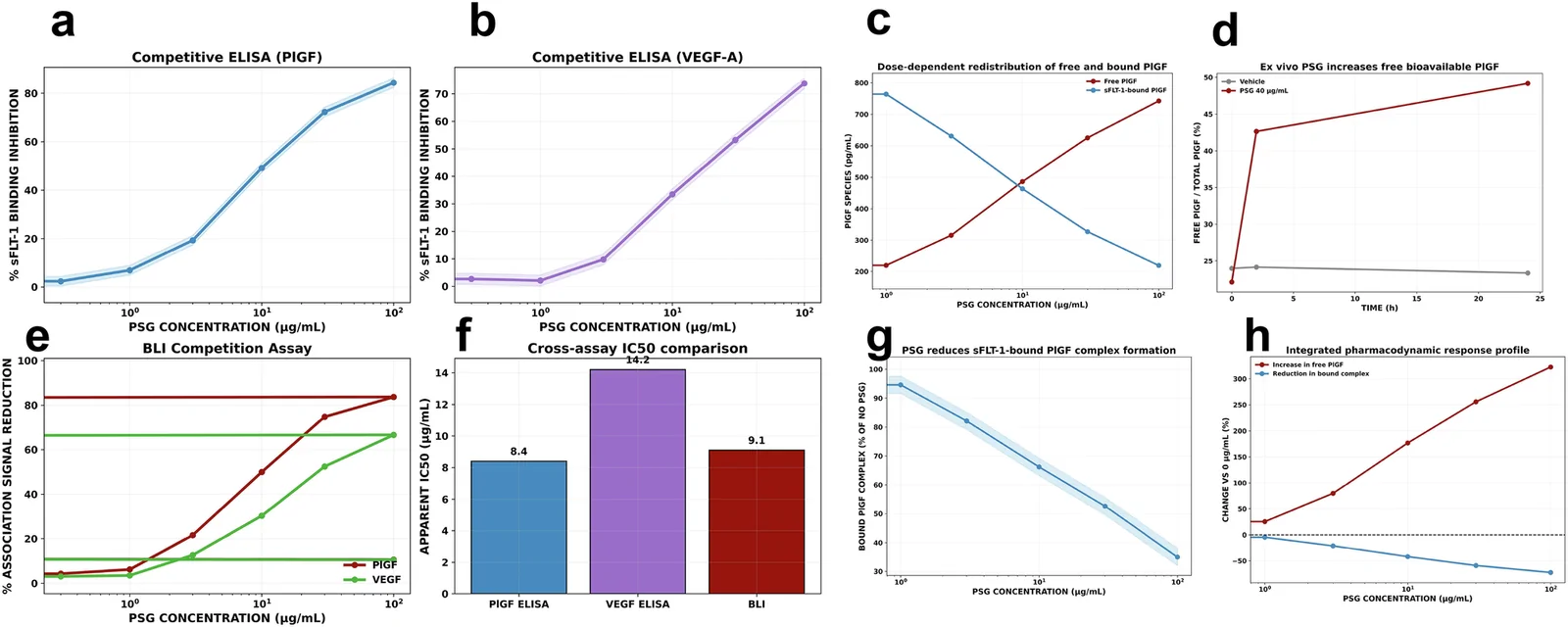
PSG disrupts sFLT-1-mediated angiogenic ligand sequestration and restores free PlGF bioavailability. Competitive ELISA analyses showing PSG-mediated inhibition of sFLT-1 binding to PlGF (**a**) and VEGF-A (**b**) across increasing PSG concentrations. BLI-based competition assay showing reduction in sFLT-1 association signals for PlGF and VEGF following PSG treatment is shown in (**c**). Comparative apparent IC50 values derived from PlGF ELISA, VEGF ELISA, and BLI competition assays are shown in (**d**). Dose-dependent redistribution of free and sFLT-1-bound PlGF species following PSG treatment is shown in (**e**). Ex vivo analysis of free bioavailable PlGF following PSG exposure over time is shown in (**f**). Quantification of sFLT-1-bound PlGF complex formation following PSG treatment is shown in (**g**). Integrated pharmacodynamic response analysis showing changes in free PlGF and reduction in bound complex formation across PSG concentrations is shown in (**h**). Data are presented as mean ± SEM

Having established direct PSG-sFLT-1 interactions through orthogonal biophysical assays (Fig. 5), we next investigated whether PSG functionally alters sFLT-1-mediated sequestration of angiogenic ligands. Competitive ELISA analyses demonstrated dose-dependent inhibition of sFLT-1 interactions with both PlGF and VEGF-A following PSG treatment (Fig. 6a, b). Similar findings were observed using BLI-based competition assays, which showed progressive reductions in sFLT-1 association signals for both ligands across increasing PSG concentrations (Fig. 6c). Cross-platform IC50 analyses demonstrated comparable inhibitory activity across ELISA- and BLI-derived interaction measurements (Fig. 6d). PSG treatment additionally induced redistribution of free and bound PlGF species, characterized by progressive increases in free PlGF together with reductions in sFLT-1-bound PlGF complexes (Fig. 6e, g). Ex vivo analyses further demonstrated increased levels of free bioavailable PlGF following PSG exposure over time (Fig. 6f). Integrated pharmacodynamic profiling showed coordinated increases in free PlGF accompanied by progressive reductions in ligand-bound complex formation across PSG concentrations (Fig. 6h).

### Maternal adverse events and safety outcomes following PSG administration in preeclampsia

Table 1 summarizes maternal adverse events and severe adverse events reported during administration of pear seed glycosides (PSG) or placebo in participants with preeclampsia. Overall, PSG administration was well tolerated, with no reported cases of heart failure, stroke, dialysis, myocardial infarction, or end-stage organ dysfunction in either treatment group. Several commonly reported pregnancy-associated symptoms, including cough, diarrhea, nausea, swelling, fatigue, and fever, occur at comparable or lower frequencies in PSG-treated participants relative to placebo controls. Chloasma was more frequently observed in the PSG group, whereas constipation remained the most reported non-serious adverse event in both groups. Importantly, severe complications associated with progressive maternal deterioration were infrequent across the study population. Seizure activity and pulmonary edema were observed only in the placebo group, while oligurea occurred at similar frequencies in both treatment arms. Collectively, these findings indicate that oral PSG administration alongside standard clinical care did not reveal evidence of major treatment-emergent safety concerns within the monitored study period.

**Table 1:** Safety and tolerability of PSG in participants with preeclampsia

	Placebo (*n* = 31)	PSG (*n* = 25)
**Maternal no**.		
Cough	3	2
Wheezing	0	1
Diarrhea	4	2
Chloasma	8	21
Headache	3	4
Heartburn	5	2
Swelling	4	1
Nausea	3	2
Constipation	24	17
Fatigue	12	8
Fever	7	2
Heart failure	0	0
Stroke	0	0
Seizure	1	0
Oligourea	4	4
Dialysis	0	0
Myocardial infarction	0	0
Pulmonary edema	2	0
End-stage organ dysfunction	0	0

### Exploratory clinical evaluation of PSG in human preeclampsia

**Fig. 7:**
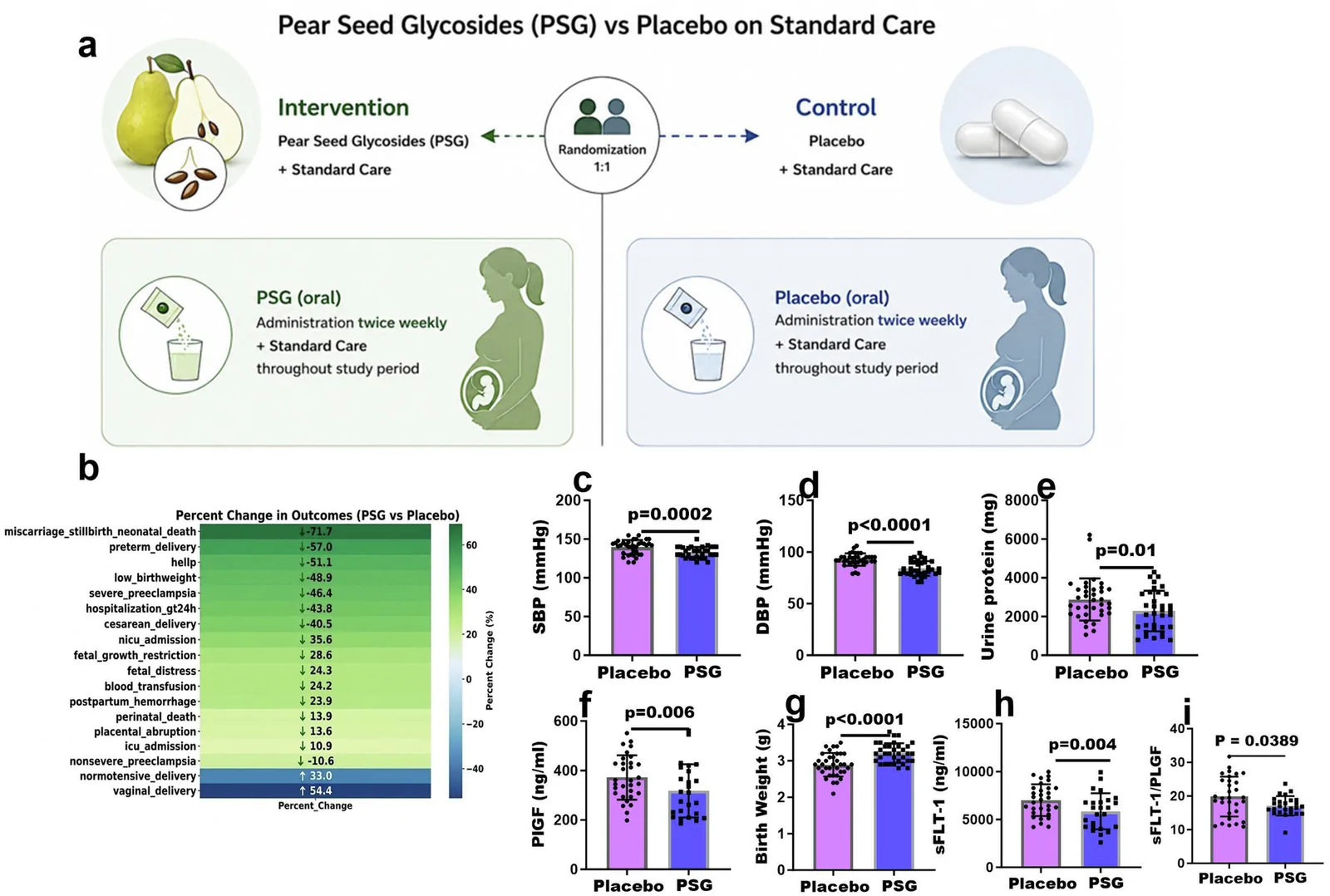
Exploratory clinical evaluation of pear seed glycosides (PSG) in preeclampsia. **a** Schematic overview of the randomized, double-blind exploratory clinical study in which participants with preeclampsia received oral PSG or placebo twice weekly alongside standard care. **b** Heatmap summarizing the percentage change in maternal and neonatal clinical outcomes in the PSG group relative to placebo. Downward arrows indicate reductions in adverse outcomes, whereas upward arrows indicate increases in favorable outcomes. Maternal systolic blood pressure (SBP; **c**), diastolic blood pressure (DBP; **d**), urine protein (**e**), circulating placental growth factor (PlGF; **f**), birth weight (**g**), circulating soluble fms-like tyrosine kinase-1 (sFLT-1; **h**), and the sFLT-1/PlGF ratio (**i**) in placebo- and PSG-treated participants. Data are presented as mean ± SEM. Statistical significance was determined using an unpaired two-tailed Student’s *t*-test

Following mechanistic and preclinical studies demonstrating PSG-mediated restoration of angiogenic signaling and functional antagonism of sFLT-1, we next performed an exploratory clinical evaluation of PSG in participants with preeclampsia receiving standard clinical care. Participants were randomized to receive either oral PSG or placebo in addition to routine management during the study period (Fig. 7a). Comparative outcome analyses demonstrated lower frequencies of multiple adverse maternal and fetal outcomes in PSG-treated participants relative to placebo-treated controls, including reductions in severe preeclampsia-associated complications, preterm delivery, fetal growth restriction, and neonatal adverse events (Fig. 7b). PSG administration was additionally associated with modulation of clinical and angiogenic parameters measured during the study period (Fig. 7c–f). Collectively, these findings extend the mechanistic and preclinical observations into a translational clinical setting and support further investigation of PSG as a candidate adjunctive therapeutic strategy in preeclampsia.

## Discussion

Preeclampsia remains difficult to manage because current treatment is largely directed toward maternal stabilization rather than reversal of the underlying placental and endothelial pathology. Antihypertensive agents such as labetalol, nifedipine, and hydralazine reduce blood pressure but do not directly correct anti-angiogenic imbalance [25, 26]. Magnesium sulfate reduces seizure risk, while aspirin is most useful as prophylaxis when initiated early in high-risk pregnancies [27, 28]. Once preeclampsia is established, definitive management still often depends on timing delivery against maternal and fetal risk [29–31]. Against this background, therapies that could safely modulate endothelial dysfunction, inflammation, and angiogenic imbalance remain an important unmet need.

In this study, pear seed-derived glycosides were evaluated across cellular, preclinical, biophysical, and exploratory clinical systems. The data suggest that pear seed extract reduces endothelial migratory, inflammatory, and fibronectin-remodeling responses in TGFβ-stimulated HUVECs, and that treatment in an L-NAME rat model is associated with improvement in maternal blood pressure, vascular responsiveness, inflammatory markers, placental architecture, and angiogenic indices. These findings are consistent with the broader concept that correcting endothelial stress and angiogenic disequilibrium may be therapeutically relevant in preeclampsia, although the animal model cannot fully reproduce the clinical heterogeneity of human disease [32–34]. Supporting pilot studies demonstrated comparable efficacy across oral, intravenous, and intraperitoneal administration, together with biological activity after disease establishment and early modulation of the sFLT-1/PlGF ratio preceding measurable hemodynamic improvement. A key aspect of the study is the bioactivity-guided narrowing of crude pear seed extract to a glycoside-enriched fraction. This is important because botanical mixtures are difficult to interpret, standardize, and regulate. The fractionation data indicate that the glycoside-rich component carries the strongest activity across maternal blood pressure and fetal growth readouts, providing a rationale for mechanistic investigation of PSG rather than continued reliance on unfractionated extract. The most mechanistically relevant finding is that PSG appears to engage the sFLT-1 axis. Excess sFLT-1 is a central anti-angiogenic mediator in preeclampsia because it binds PlGF and VEGF, thereby reducing pro-angiogenic signaling at the maternal endothelium [35, 36]. Several therapeutic strategies in development, including apheresis-based sFLT-1 removal, RNA-based approaches to reduce placental sFLT-1 production, and agents aimed at restoring angiogenic balance, are based on this biology [13, 14]. Within that landscape, PSG is positioned not as a replacement for established obstetric management, but as a possible adjunctive approach that may functionally interfere with sFLT-1-mediated ligand sequestration. The SPR/BLI, MST, competition, free-PlGF, and complex-displacement assays provide a coherent biochemical framework supporting this possibility. The downstream HUVEC signaling data further suggest that PSG may restore VEGFR2/KDR, AKT, and ERK activity suppressed by sFLT-1 exposure. The exploratory clinical component provides preliminary safety and translational context but should be interpreted cautiously. PSG administration alongside standard care did not reveal major treatment-emergent safety concerns within the monitored period, and the clinical outcome patterns appear directionally consistent with the preclinical and mechanistic findings. Although both circulating sFLT-1 and PlGF were reduced following PSG administration, the proportionally greater reduction in sFLT-1 resulted in an overall improvement in the clinically relevant sFLT-1/PlGF ratio, the biomarker most widely used to assess angiogenic balance in preeclampsia. The basis for the observed reduction in circulating PlGF remains uncertain, particularly given the contrasting increase in PlGF observed in the preclinical model, and warrants further mechanistic investigation in larger clinical cohorts. Worthy of note, the current trial was early-phase, modest in size, and not powered to establish clinical efficacy. Larger randomized trials with prespecified endpoints, rigorous adverse-event adjudication, pharmacokinetic assessment, and longer maternal-neonatal follow-up will be required. Overall, these findings support further investigation of PSG as a candidate adjunctive strategy targeting angiogenic dysfunction in preeclampsia, while emphasizing that its clinical role remains provisional.

## Limitations

Several limitations should be considered when interpreting the present findings. First, although the study integrates endothelial, preclinical, biophysical, pharmacodynamic, and exploratory clinical datasets into a coherent mechanistic framework, PSG remains a glycoside-enriched fraction rather than a fully resolved single molecular entity. Further analytical characterization will therefore be important to define the precise active constituents and improve standardization across preparations. Second, while the orthogonal binding and competition assays support functional interaction between PSG and sFLT-1, the precise structural interface and binding determinants remain to be established. Additional structural and biophysical studies may help clarify whether PSG directly occupies ligand-binding regions or modulates sFLT-1 conformation indirectly. Third, the L-NAME model reproduces several vascular and angiogenic features of preeclampsia but cannot fully capture the immunologic, placental, and clinical complexity of human disease. Nevertheless, the consistency observed across endothelial, animal, and translational datasets strengthens the biological plausibility of the findings. Finally, the clinical component was designed primarily as an exploratory early-phase assessment of safety, tolerability, and preliminary biological activity rather than a definitive efficacy trial. Larger multicenter studies with longer follow-up, predefined clinical endpoints, and pharmacokinetic evaluation will therefore be important to further define the therapeutic potential and translational applicability of PSG in preeclampsia.

## Supplementary information

Supplementary Figure 1

Supplementary Figure 2

Supplementary Figure 3

Supplementary information

## Data Availability

All data are available from the corresponding author upon reasonable request. Please refer to the data management and sharing plan in the attached protocol document.
