# Supplementary material for: Glycoside fraction from pear seed functionally antagonizes sFLT-1 and restores angiogenic signaling in preeclampsia
Source: Hypertens Res. 2026 Sep 1;49(11):3177–93. doi: 10.1038/s41440-026-02770-x (PMC13736672; doi:10.1038/s41440-026-02770-x)

Uncropped western blot files

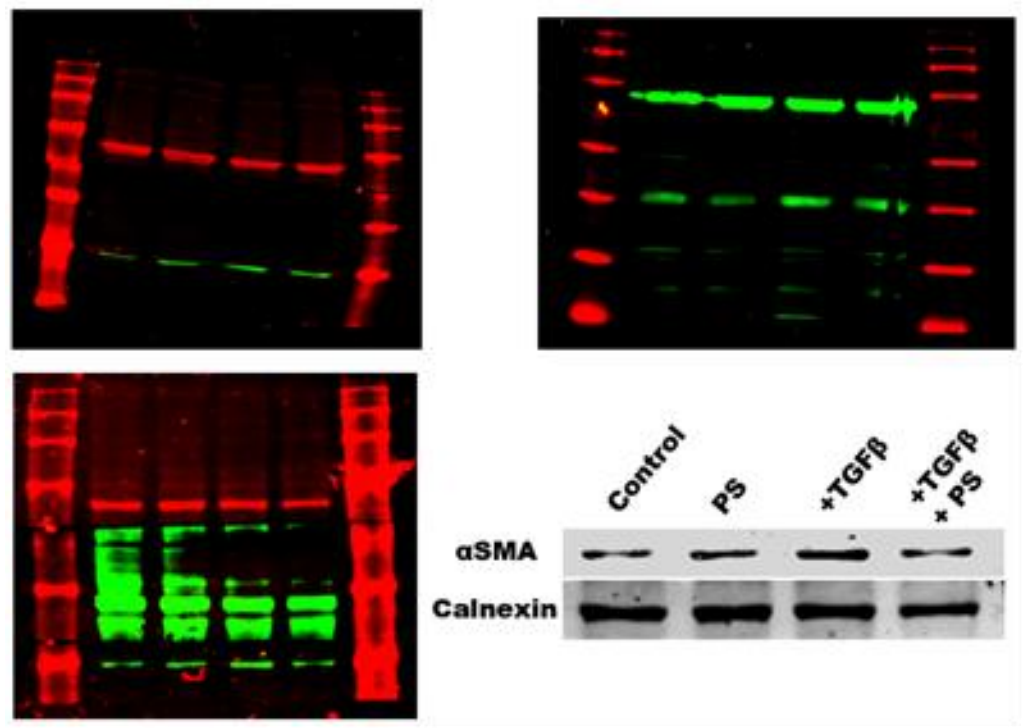

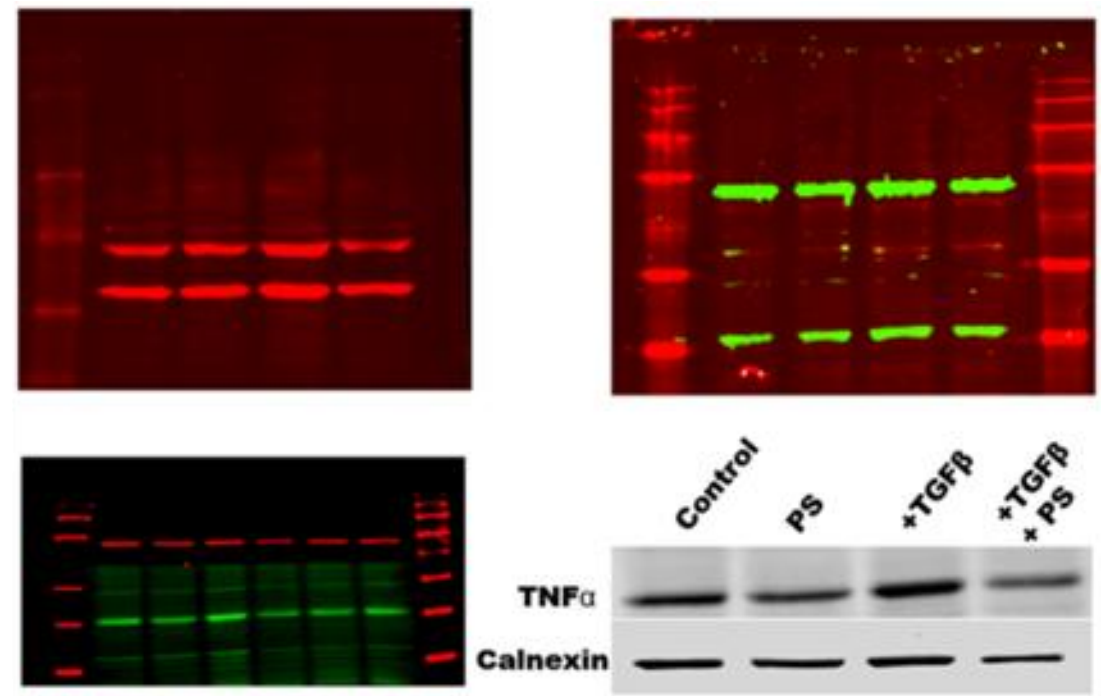

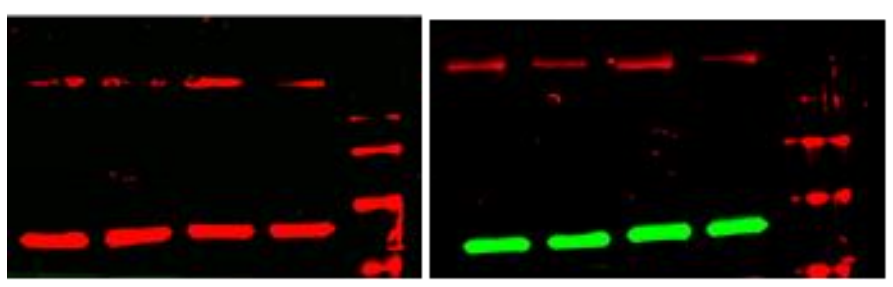

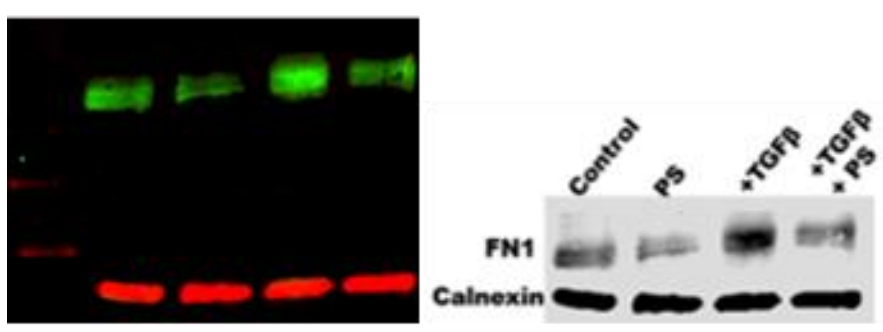

Supplement: Supplementary file 1 [file 41440_2026_2770_MOESM4_ESM.pdf]
